# Supplementary material for: Deoxynivalenol Induces Local Inflammation and Lesions in Tissues at Doses Recommended by the EU
Source: Int J Mol Sci. 2024 Sep 10;25(18):9790. doi: 10.3390/ijms25189790 (PMC11432646; doi:10.3390/ijms25189790)
Supplement: Supplementary file 1 [file ijms-25-09790-s001.zip › ijms-3149358 supplementary-material.pdf]

International Journal of
Molecular Sciences

MDPI

Supplementary material

Deoxynivalenol, induces local inflammation and lesions in tissues at doses recommended by the EU

Alix Pierron [1,†], Luciana C. Balbo [2], Laura Soler [1], Philippe Pinton [1], Sylvie Puel [1], Joëlle Laffitte [1], Mickaël Albin [1], Ana-Paula F. R. Loureiro Bracarense [2], Maria A. Rodriguez [3] and Isabelle P. Oswald [1,*]

1 Toxalim (Research Centre in Food Toxicology), Université de Toulouse, INRAE, ENVT, INP-Purpan, UPS, 31027 Toulouse, France; alix.pierron@envt.fr (A.P.); laura.soler@inrae.fr (L.S.); philippe.pinton@inrae.fr (P.P.); sylvie.puel@inrae.fr (S.P.); jlaffitte@posteo.net (J.L.); mickael.albin@inrae.fr (M.A.); isabelle.oswald@inrae.fr (I.P.O.)

2 Laboratory of Animal Pathology, Universidade Estadual de Londrina, Londrina 86057-970, Brazil; luciana.cbalbo@uel.br (L.C.B.); anapaula@uel.br (A.-P.F.R.L.B.)

3 Olmix, 56580 Bréhan, France; mrodriguez@olmix.com

* Correspondence: isabelle.oswald@inrae.fr

† Current address:
National Vet School of Toulouse (ENVT), INRAE, UMR 1225, IHAP, Université de Toulouse, 31300 Toulouse, France.

Abstract: The mycotoxin deoxynivalenol (DON) is frequently present in cereals at low levels, resulting in its occurrence in food and feed. DON has been proven to alter the immune response and induce inflammation in all species, with pigs exhibiting heightened sensitivity and exposure. However, no study has yet evaluated the effects of exposure to DON at the recommended levels in pig feed. In two separate trials, piglets were subjected to control feed or feed contaminated with a low level of purified DON (0.83 mg/kg feed in trial 1 and 0.85 mg/kg feed in trial 2) for either three weeks (trial 1) or two weeks (trial 2). Additionally, a group of animals exposed to 2.85 mg/kg feed of DON was included as a positive control in Trial 1. The impact of DON on porcine tissues (intestine, liver, and spleen) was evaluated through histological and qPCR analyses of immune-related genes. Additionally, biochemical analyses and acute-phase proteins were examined in plasma samples. Lesions were identified in the intestine (jejunum and ileum), the liver, and the spleen of pigs receiving diets contaminated with low and high concentrations of DON. The low level of DON also resulted in impaired expression of genes associated with intestinal barrier integrity, intestinal immune responses, and liver function. In conclusion, the results of the two trials demonstrate the impact of DON exposure even at doses below the recommended level of 0.9 mg/kg feed set by the European Union. This suggests that the current recommended level should be reconsidered to ensure the optimal health and well-being of pigs.

Keywords: mycotoxins; toxicity; DON; histology; immunity-related genes

Citation: To be added by editorial staff during production.

Academic Editor: Firstname Lastname

Received: date
Revised: date
Accepted: date
Published: date

Copyright: © 2024 by the authors. Submitted for possible open access publication under the terms and conditions of the Creative Commons Attribution (CC BY) license (https://creativecommons.org/licenses/by/4.0/).

A.

		1	2
RAW MATERIALS	UNITS	VALUE	VALUE
WHEAT (10.4% MAT)	%	42,15	41,56
WHEAT	%		0,10
BARLEY	%	35,00	35,00
RAPESEED MEAL 46.2% MAT	%	15,00	
SOYBEANMEAL HIPRO 48/3.5	%		14,33
RAPESEED MEAL	%	3,50	3,50
HUILE DE TOURNESOL	%	0,75	
SOYBEAN OIL	%		1,18
SALT	%	0,54	0,54
DICALCIUMFOSFAAT	%	0,84	0,84
LIMESTONE	%	0,43	1,19
L-LYSIN HCL	%	0,62	0,65
METHIONINE (DL)	%	0,20	0,22
L-THREONINE	%	0,26	0,26
TRYPTOPHAN (L)	%	0,05	0,05
VALINE	%	0,14	0,17
NATUPHOS 5000G	%	0,02	
NATUPHOS E 10000G	%		0,01
CP2017 - PIGLET 2A INRA E21 0.5%	%	0,50	
PREMIX Growing Pigs 0.4% (TWM)	%		0,40
TOTAL	%	100,00	100,00
NUTRIENTS	UNITS	VALUE	VALUE
Protein	%	17,0	17,0
Fat	%	2,3	2,7
Fiber	%	4,0	3,5
Ash	%	4,6	5,2
NDF	%	13,4	
Phosphores	%	0,53	0,51
Phosphores d	%	0,34	0,31
Calcium	%	0,62	0,8
Sodium	%	0,20	0,2
Energy Netto	kcal/kg	2311	2349,0
Lys	%	1,23	1,3
Lys d	%	1,12	1,1
M+C d / Lys d	%	60,0	59,00
Thr d / Lys d	%	64,0	61,00
Try d / Lys d	%	20,0	19,0
Iso d / Lys d	%	48,2	47,0
Val d / Lys d	%	68,0	69,0

B.

Trial	Duration		Control (ppm)	Low DON (ppm)	High DON (ppm)
1	3 weeks	***DON***	**0.225**	**0.850**	**2.850**
		D3G	0.040	0.030	0.040
		Nivalénol	0.010	0.010	0.015
		15-O-acétyl-DON	0.010	0.010	0.015
		Zearalenone	0.010	-	-
		Ergotamine	0.015	-	0.010
		Ergocristine	-	-	0.015
		Ergosine	-	-	0.040
2	2 weeks	***DON***	**0.035**	**0.825**	-

C.

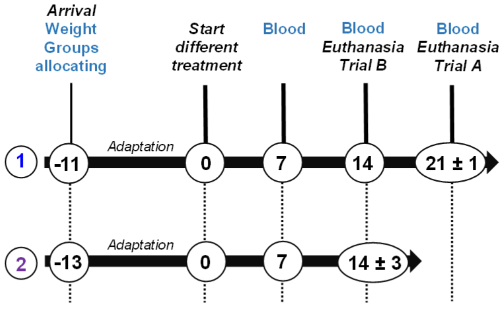

Figure S1: Experimental details of the animal trials. A) Animals were exposed to a second age formulated diet during all the experiment. A same diet formulation was calculated for the two trials but furnished by two different feed producers, inducing small changes. B) Real concentration of deoxynivalenol and some others mycotoxins or metabolites above their detection level obtained in the different regiment for the Trial 1 and the Trial 2. C) Experimental scheme for the Trial 1 and trial 2. for both trials, animals arrived at weaning age then passed by an adaptation phase of 11 or 13 days. Then were exposed to the different regiments during three weeks for the Trial 1 and two weeks for the Trial 2. Every week, animals were weighed and their blood was collected. At the end of the experiment they were euthanized and different organs were collected.

Table S1: Primer sequences used for RT-qPCR analysis (F: Forward, R: reverse).

Gene Symbol	Gene Name	Primer sequence	References
HMBS	Hydroxymethylbilane synthase	F: AGGATGGGCAACTCTACCTG	ENSSSCT00000060506.2
		R: GATGGTGGCCTGCATAGTCT	Pierron et al. 2022
B2M	Beta-2-Microglobulin	F: CTGCTCTCACTGTCTGG	ENSSSCT00005043116.1
		R: TTCAGGTAATTTGGCTTTCC	Pierron et al. 2022
RPL32	Ribosomal protein	F: AGTTCATCCGGCACCAGTCA	NM_001001636
		R: GAACCTTCTCCGCACCCTGT	Pinton et al. 2010
TOP2B	DNA Topoisomerase II Beta	F: AAGGGCGAGAGGTCAATGAT	AY550069
		R: ACATCTTCTCGTTCTTGCGC	Park et al. 2015
IL1 beta	Interleukin 1 beta	F: ATGCTGAAGGCTCTCCACCTC	NM_214055
		R: TTGTTGCTATCATCTCCTTGCAC	Bracarence et al. 2020
Il1 alpha	Interleukin 1 alpha	F: GCCAATGACACAGAAGAAGA	NM_214029
		R: ATGCACTGGTGGTTGATG	Pierron et al. 2022
CXCL8	Interleukine 8	F: GCTCTCTGTGAGGCTGCAGTTC	NM_213867
		R: AAGGTGTGGAATGCGTATTTATGC	Cano et al. 2013
IL10	Interleukine 10	F: GGCCCAGTGAAGAGTTTCTTTC	NM_214041
		R: CAACAAGTCGCCCATCTGGT	Cano et al. 2013
IFNgamma	Interferon gamma	F: TGGTAGCTCTGGGAAACTGAATG	NM_213948
		R: GGCTTTGCGCTGGATCTG	Cano et al. 2013
TNFalpha	Tumor necrosis factor alpha	F: ACTGCACTTCGAGGTTATCGG	NM_214022
		R: GGCGACGGGCTTATCTGA	Cano et al. 2013
IL12p40	Interleukin 12 subunit 40	F: GGTTTCAGACCCGACGAACTCT	NM_214013
		R: CATATGGCCACAATGGGAGATG	Cano et al. 2013
TGFB1	Transforming Growth Factor 1	F: GGATACCAACTACTGCTTCAG	ENSSSCT00000036469.2
		R: GGTTCATGAATCCACTTCCA	This Study
NFKB1	Nuclear Factor Kappa B	F: CCTCCACAAGGCAGCAAATAG	ENSSSCT00000033438
		R: TCCACACCGCTGTCACAGA	This Study
IGFB1	Insulin-like Growth Factor 1	F: TTCAGTTCGTGTGCGGAGAC	NM_214256.1
		R: CGTACCCTGTGGGCTTGTTG	This Study
MUC2	Mucin 2	F: GCAGCCTGTGCGAGGAA	XM_003122394.1
		R: TGTCATCATACACAGTGCCTTCTG	Pinton et al. 2015
CCK	Cholecystokinin	F: AAAGCACCTTCTGGCCGAGT	NM_214237
		R: GGTCACTTATTCTGTGGCTGGG	This Study
SOD1	Superoxyde Dismutase 1	F: ATCATGGATTCCATGTCCATCAG	NM_001190422
		R: GGACCTGCACTGGTACAGCC	This Study
SOD2	Superoxyde Dismutase 2	F: AGTACCAGGAGGCGCTGAAA	NM_214127
		R: GCAGAGCTACCTGAGCTGTAACATC	This Study
ABCA1	ATP-binding cassette, sub-family A member 1	F: ACCTGGTGAAGACCTATGTGCAG	ENSSSCT00000027427
		R: CAGATCTTGTTCTTTAAGCTTTTGGC	This Study
Casp3	Procaspase 3	F: ATAATAAGAACTTTGATAAAAATACCGGAATG	NM_214131
		R: TCCACATCTGTACCAGATCGACAT	This Study
Casp9	Caspase 9	F: CACACCCAGTGACATCTTT	AB020979.1
		R: CAAAGCGACTGGATGAAC	Hasuda et al. 2022

Author Contributions: Conceptualization, I.P.O. and M.A.R.; methodology, A.P., P.P., L.S., and M.A.; formal analysis, A.P., L.C.B., J.L., S.P., and A.-P.F.R.L.B.; data curation, A.P.; writing—original draft preparation, A.P.; writing—review and editing, A.P. and I.P.O.; supervision, I.P.O. and M.A.R.; project administration, I.P.O.; funding acquisition, I.P.O. All authors have read and agreed to the published version of the manuscript.

Funding: This study was partially funded by the DONAlgue project funded by the company Olmix.

Institutional Review Board Statement: The two animal studies were carried out in accordance with the European Directive on the protection of animals used for scientific purposes (Directive 2010/63/EU) and approved by the Ethics Committee for Animal Experiments Toxcomethique n°86 (TOXCOM/255/API).

Data Availability Statement:

Conflicts of Interest: The authors declare no conflicts of interest. The funders had no role in the collection, analyses, or interpretation of data, nor in the writing of the manuscript. "Author Maria A. Rodriguez is employed by the company OLMIX. The remaining authors declare that the research was conducted in the absence of any commercial or financial relationships that could be construed as a potential conflict of interest".

References

18. Hasuda, A.L.; Person, E.; Khoshal, A.K.; Bruel, S.; Puel, S.; Oswald, I.P.; Bracarense, A.P.F.R.L.; Pinton, P. Deoxynivalenol induces apoptosis and inflammation in the liver: Analysis using precision-cut liver slices. *Food Chem. Toxicol.* **2022**, *163*, 112930.
27. Bracarense, A.P.F.L.; Pierron, A.; Pinton, P.; Gerez, J.R.; Schatzmayr, G.; Moll, W.D.; Zhou, T.; Oswald, I. P. Reduced toxicity of 3-epi-deoxynivalenol and de-epoxy-deoxynivalenol through deoxynivalenol bacterial biotransformation: In vivo analysis in piglets. *Food Chem. Toxicol.* **2020**, *140*, 111241.
43. Pierron, A.; Neves, M.; Puel, S.; Lippi, Y.; Soler, L.; Miller, J. D.; Oswald, I.P. Intestinal toxicity of the new type A trichothecenes, NX and 3ANX. *Chemosphere.* **2022,** *288*, 132415.
44. Pinton P, Braicu C, Nougayrede JP, Laffitte J, Taranu I, Oswald IP. Deoxynivalenol impairs porcine intestinal barrier function and decreases the protein expression of claudin-4 through a mitogen-activated protein kinase-dependent mechanism. *J Nutr.* **2010**, *140,* 1956-62.
45. Park, S.J.; Kwon, S.G.; Hwang, J.H.; Park, D.H.; Kim, T.W.; Kim, C.W. Selection of appropriate reference genes for RT-qPCR analysis in Berkshire, Duroc, Landrace, and Yorkshire pigs. *Gene***. 2015**, *558*, 152–158.
46. Cano, P.M.; Seeboth, J.; Meurens, F.; Cognie, J.; Abrami, R;. Oswald, I.P.; Guzylack-Piriou, L. Deoxynivalenol as a new factor in the persistence of intestinal inflammatory diseases: an emerging hypothesis through possible modulation of Th17-mediated response *PLoS One.* **2013**, *8,* e53647.
47. Pinton, P.; Graziani, F.; Pujol, A.; Nicoletti, C.; Paris, O.; Ernouf, P.; Di Pasquale, E.; Perrier, J.; Oswald, I.P.; Maresca, M. Deoxynivalenol inhibits the expression by goblet cells of intestinal mucins through a PKR and MAP kinase dependent repression of the resistin-like molecule β. *Mol. Nutr. Food Res.* **2015**, *59,* 1076-87.

Disclaimer/Publisher's Note: The statements, opinions and data contained in all publications are solely those of the individual author(s) and contributor(s) and not of MDPI and/or the editor(s). MDPI and/or the editor(s) disclaim responsibility for any injury to people or property resulting from any ideas, methods, instructions or products referred to in the content.
